# Characterization of humoral immune responses and degree of protection induced by influenza vaccine in cotton rats: Effects of low vaccine dose and single vs booster vaccination

**DOI:** 10.1002/iid3.303

**Published:** 2020-04-22

**Authors:** Yoshita Bhide, Wei Dong, Tjarko Meijerhof, Jacqueline de Vries‐Idema, Hubert G. Niesters, Anke Huckriede

**Affiliations:** ^1^ Department of Medical Microbiology and Infection Prevention, University Medical Center Groningen University of Groningen Groningen The Netherlands

**Keywords:** cotton rats, humoral immune response, lung viral load, respiration rate, virus challenge, whole inactivated virus (WIV) influenza vaccine

## Abstract

**Introduction:**

Cotton rats are a suitable model for the study of influenza disease symptoms and responses to influenza vaccination. We have previously shown that two immunizations with 15 µg whole inactivated virus (WIV) influenza vaccine could completely protect animals from infection with the H1N1pdm09 virus.

**Methods:**

To further explore the cotton rat model, we here investigated the protective potential of a single intramuscular immunization and of prime/boost intramuscular immunizations with a low amount of antigen.

**Results:**

A single intramuscular immunization with doses more than or equal to 0.5 µg WIV reliably evoked antibody responses and doses more than or equal to 1 µg protected the animals from virus replication in the lungs and from severe weight loss. However, clinical symptoms like an increased respiration rate were still apparent. Administration of a booster dose significantly increased the humoral immune responses but did not or only moderately improved protection from clinical symptoms.

**Conclusion:**

Our data suggest that complete and partial protection by influenza vaccines can be mimicked in cotton rats by using specific vaccination regimens.

## Graphical abstract

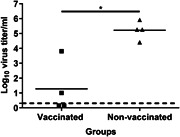

**Abbreviation**
WIV: whole inactivated virus influenza vaccine

## 1 INTRODUCTION

Cotton rats (*Sigmodon hispidus*) have been used as a small animal model for respiratory virus infections such as human parainfluenza virus, respiratory syncytial virus, measles virus, human metapneumovirus, as well as for the study of influenza virus infection and pathogenesis.^1^, ^2^, ^3^, ^4^, ^5^, ^6^, ^7^ All these respiratory viruses can easily replicate in cotton rats and induce pathogenesis similar to that in humans. Unlike mice, cotton rats are susceptible to clinical isolates of influenza virus without prior adaptation of the virus.^4^, ^8^, ^9^ Indeed, cotton rats have been infected successfully with a broad range of clinical influenza virus strains.^4^, ^6^, ^10^ Upon infection, cotton rats show easily quantifiable disease symptoms like increased respiration rate, weight loss, and hypothermia.^11^, ^12^

Despite the positive results with cotton rats as a model for influenza infection, there is so far limited information on the performance of influenza vaccines in cotton rats. Early studies evaluated the effect of infection‐induced immunity on subsequent challenge with homologous or heterologous virus strains.^13^, ^14^ Later, the studies were extended to ultraviolet‐inactivated virus, seasonal trivalent split vaccine, and more recently recombinant adenovirus vaccines and AS03‐adjuvanted A(H1N1)pdm09 pandemic influenza vaccine.^11^, ^12^, ^15^, ^16^, ^17^ Some of these studies report on a reduced increase in the respiration rate in immunized vs naïve cotton rats upon challenge.^13^, ^15^ Some studies investigated additional parameters like lung virus titers and lung pathology and/or determined induction of antibodies. However, none of the studies gives a complete description of clinical symptoms like changes in weight and temperature. Moreover, none of the studies describes the evaluation of vaccines against H1N1pdm virus, a clinically highly relevant virus that is still in circulation.

We have shown earlier that through intramuscular or pulmonary immunization with a high vaccine dose, 15 µg whole inactivated virus (WIV) vaccine, cotton rats can be protected completely against replication of H1N1pdm virus in the lungs and against the development of severe clinical symptoms.^18^ To get more insight into the model, we here assessed the effect of vaccine dose and vaccination regimen on humoral immune responses and the protective potential in detail.

To this end, we immunized cotton rats once or twice with low doses of a WIV influenza vaccine derived from an H1N1pdm09 vaccine strain and challenged the animals with a clinical isolate of H1N1pdm09 virus. Antibody titers in immunized animals correlated with vaccine dose and were boosted by a second immunization. Upon challenge, the lung virus titers on days 1 and 3 post‐challenge were strongly reduced as compared with titers in control animals. Yet, immunization had limited effects on clinical symptoms like tachypnea and disease parameters or on induction of cytokines and Mx1 in the lungs. We conclude that low or moderate vaccine doses of WIV could protect outbred cotton rats partly from the lethal virus infection. As such, the tested vaccination regimens allow studying different scenarios as encountered in humans.

## 2 MATERIALS AND METHODS

### 2.1 Vaccine and virus

NIBRG‐121, a vaccine strain produced from the A/California/7/2009 virus, was obtained from NIBSC (Potters Bay, UK) and grown in embryonated chicken eggs followed by purification using sucrose gradient centrifugation. The virus was inactivated by overnight treatment with 0.1% β‐propiolactone (Acros Organics, Geel, Belgium) in citrate buffer (125 mM sodium citrate, 150 mM sodium chloride, pH 8.2) at 4°C to produce WIV vaccine. Inactivation was followed by dialysis against HNE buffer (145 mM NaCl, 5 mM HEPES, 1 mM EDTA, pH 7.4, sterilized by autoclaving). Inactivation of the virus was verified by inoculation of MDCK cells and the amount of protein was determined by micro‐Lowry assay.

A clinical isolate of H1N1pdm (isolate E9‐6714) was provided by the Department of Clinical Virology, UMCG. The virus, to be called A/Cal/Gro in the following, was diagnosed by quantitative polymerase chain reaction (PCR) as being similar to A/California/7/2009 virus. The virus was further amplified on MDCK cells, titrated in cotton rats and was used as challenge virus. Virus titer was determined by TCID50 titration.^19^ Whole inactivated A/PR/8/34 (H1N1) and the X‐31 (H3N2, reassortant strain of A/Aichi/68 and A/PR/8/34 viruses) used for determination of cross‐reactive immunoglobulin (IgG) enzyme‐linked immunosorbent assay (ELISA) were kindly provided by NIBSC.

### 2.2 Cotton rats and immunization

All animal experiments were approved by the Institutional Animal Care and Use Committee of the University of Groningen (IACUC‐RuG), The Netherlands. Outbred female cotton rats at an age of 10 to 12 weeks were purchased from Harlan Laboratories. The animals were housed in individually ventilated cages with two cotton rats per cage. All the cotton rats were injected with implantable electronic ID transponders (Bio Medic Data Systems Inc [BMDS], Seaford, DE) subcutaneously (s.c.) for individual identification and temperature measurement. The weight of the cotton rats ranged between 120 and 150 g during the challenge phase. A sample size of four animals per group was used based on literature.^13^, ^20^, ^21^

The study comprised two independent experiments, the details of which are described in Table 1.

**Table 1:** Experimental details of the two animal experiments

Experiment	1	2
Groups and vaccine	PBS control (*n* = 4)	PBS control (*n* = 8)
	0.5 µg WIV (*n* = 4)	1 µg WIV (*n* = 8)
	1 µg WIV (*n* = 4)	Nontreated control (*n* = 2)
	5 µg WIV (*n *= 4)	
Vaccination	1 dose, day 1	2 doses, day 1 and day 21
Virus challenge	5 × 10^7^ TCID50	1 × 10^7^ TCID50
	(day 30)	(day 51)

Abbreviations: PBS, phosphate‐buffered saline; WIV, whole inactivated virus.

Cotton rats were vaccinated via the intramuscular route with 100 µL vaccine distributed over the hind limbs. Four weeks after the single immunization in experiment 1 or the second immunization in experiment 2, cotton rats were challenged with A/Cal/Gro, 5 × 10^7^ TCID50 in experiment 1 (on day 30) and 1 × 10^7^ TCID50 in experiment 2 (on day 51). The virus in a volume of 100 µL was distributed over both the nostrils using a pipette. Both vaccination and challenge were carried out under 5% isoflurane/O_2_ anesthesia. The two nontreated control animals in experiment 2 received neither vaccination nor challenge.

### 2.3 Assessment of clinical symptoms and sample collection

After challenge, cotton rats were followed daily for determination of changes in weight, temperature and respiration rate for 3 days in the first experiment and 10 days in the second experiment. Animals were weighed by catching them into a preweighed cardboard roll. Weight loss of 10% in 1 day or 15% from the day of challenge were considered as criteria for the humane endpoint. Ten days post‐challenge, the remaining cotton rats were killed.

The respiration rate was measured by plethysmography, as described previously.^22^ Briefly, the animal was placed in a 1500 mL air‐tight but transparent tube of a whole‐body plethysmograph, which was connected to a pressure transducer. The frequency of pressure changes inside the tube was recorded and displayed as breaths per minute (bpm). The mean respiration rate of an animal was then calculated from a minimum of four steady regions lasting for at least 15 seconds. For many of the animals, if they breathed at a constant rate, we even recorded the respiration for a minute or more consecutively. The maximal variation between the readings was ±5 to 10 bpm, thus about 1% to 3%. Temperature was measured while the animal was restrained in the cardboard container using a DAS‐7008/9 detector for s.c. injected electronic ID transponders (BMDS, Seaford, USA).

Blood was drawn on the day(s) of immunization, the day of challenge and the days of sacrifice. Serum was separated and stored at −20°C until assessment of IgG, microneutralization (MN) and hemagglutination inhibition (HI) antibodies. A small part of the same lung was stored in RNAlater (Qiagen, the Netherlands) for cytokine profiling by quantitative real‐time PCR (qRT‐PCR).

### 2.4 Lung virus titration

Equally sized parts of the lungs were collected in 1 ml complete EPISERF medium (100 U/mL penicillin, 100 mg/mL streptomycin, 1 M HEPES, 7.5% sodium bicarbonate, all Life Technologies BV, Bleiswijk, The Netherlands) and were homogenized, centrifuged, and the supernatants were used for determination of viral load in the lungs, as described previously.^19^ Virus amounts are represented as log_10_ titer per mL of medium. The limit of detection (LoD) was calculated using the first dilution made; negative samples were assigned a value corresponding to half of the LoD value for calculation purposes.

### 2.5 IgG and IgA enzyme‐linked immunosorbent assay

IgG ELISA was performed by coating ELISA plates (Greiner Bio‐One, Alphen a/d Rijn, The Netherlands) with 0.3 µg/well of A/PR/8, H1N1pdm or X‐31 WIV in coating buffer (17.8 mM Na_2_CO_3_, 22.5 mM NaHCO_3_, pH9.6) overnight at 37°C. ELISA was done as described previously.^23^ IgG titers were calculated as log_10_ of the reciprocal of the sample dilution corresponding to an absorbance of 0.2 at the wavelength of 492 nm. LoD was calculated using the first dilution made and the negative samples were given half the value of the LoD value.

### 2.6 MN assay and hemagglutination inhibition

Serum samples taken on the day of the second immunization, the day of challenge and 10 days post‐challenge were assessed for MN and HI antibodies against A/California/2009 virus using a previously described protocol,^23^, ^24^ respectively. Titers are presented as log_2_ HI titers for individual cotton rats. LoD was calculated using the first dilution made and the negative samples were given half the value of the LoD value.

### 2.7 Cytokine measurement by quantitative real‐time polymerase chain reaction

For determination of cytokine expression in the lungs, the lungs stored in RNAlater were homogenized using a pestle and RNA was extracted with the help of the QIAGEN RNeasy extraction mini kit (Qiagen, Hilden, Germany). Complementary DNA was synthesized with a PrimeScript RT‐PCR Kit (Takara, Westburg, Leusden, the Netherlands) using 500 ng RNA. qPCR was run with specific anti‐cotton rat primers for Mx1, Mx2, IFNγ, IFNα, IL1β, IL4, IL6, and IL12 (primer sequences, see Table S1). Primers were designed with help of the program Primer Blast, using cotton rat sequences from NCBI BLAST. The specificity of the primers was validated by checking if there was a single melt curve for all samples tested. Glyceraldehyde 3‐phosphate dehydrogenase (GAPDH) was used as a housekeeping gene. PCR cycling conditions were set as 10 minutes 95°C followed by 40 cycles of 15 seconds 95°C and 1 minute 60°C on an Applied Biosystems’ StepOnePlus real‐time PCR system. SYBR green ROX‐mix used was from Westburg (Leusden, the Netherlands).

For analysis, mean *C*_T_ values of GAPDH per sample were subtracted from the mean *C*_T_ values of the cytokine for the same animal to calculate Δ*C*_T_ values. ΔΔ*C*_T_ values were then calculated by subtracting Δ*C*_T_ of the nontreated cotton rats from Δ*C*_T_ of the vaccinated and nonvaccinated cotton rats that were challenged and killed on day 1 and day 10 post‐challenge. The fold change was then calculated. Cytokines are represented as log_2_ fold changes in vaccinated and nonvaccinated cotton rats with respect to nontreated cotton rats.

### 2.8 Statistical analysis

The statistical analysis was performed using GraphPad Prism 5 software (GraphPad Software, La Jolla, CA) with which the graphs were plotted as well. The nonparametric Mann‐Whitney *U* test was used to test if the differences between two groups, that is, vaccinated and nonvaccinated cotton rats, with respect to different parameters were significant. A *P* value of less than 0.05 was considered significant.

## 3 RESULTS

### 3.1 Systemic immune response after a single vaccination

**Figure 1:**
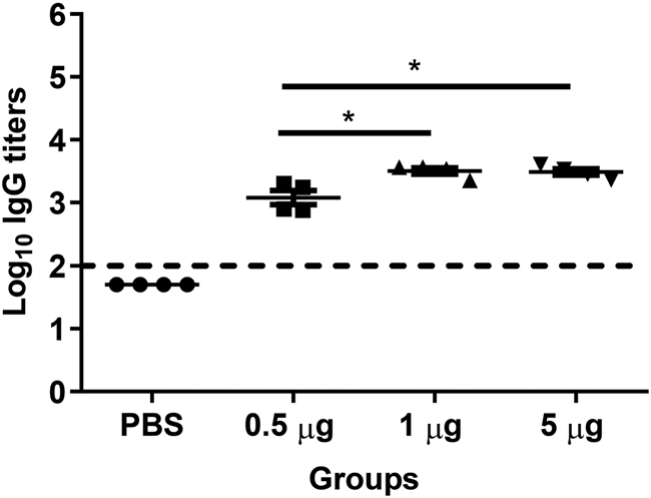
Systemic immune response after a single vaccination. Serum IgG responses were evaluated 30 days after a single immunization with the indicated amounts of WIV. IgG titers are presented as log_10_ titers for individual cotton rats with means per group (*n* = 4). LoD is indicated at 2 with a dashed line and significance is represented as *P < .05. IgG, immunoglobulin; LOD, limit of detection; PBS, phosphate‐buffered saline; WIV, whole inactivated virus

To assess the immune response induced by a single IM immunization with WIV, sera were collected on the day of immunization and the day of challenge and serum IgG titers were evaluated by ELISA. On the day of immunization, all cotton rats were sero‐negative for influenza (data not shown). On the day of challenge, all vaccinated cotton rats had developed antibodies with a titer of 10^3^ or higher. Compared with IgG titers induced by 0.5 µg WIV, significantly higher IgG titers were induced by 1 and 5 µg WIV (Figure 1), yet, the differences were rather small (about 2.5‐fold). No difference was found between the IgG titers induced by 1 and 5 µg WIV. Thus, all vaccine doses induced robust serum IgG responses upon a single vaccination.

### 3.2 Effect of a single immunization on clinical symptoms after virus challenge

We next assessed the effect of a single vaccination with low and moderate vaccine doses on disease symptoms and lung virus replication upon infection. To this end, cotton rats were i.m. injected with a single dose of either PBS, 0.5, 1, or 5 µg WIV followed by homologous challenge with a dose of 5 × 10^7^ TCID_50_ H1N1pdm 30 days later. Upon challenge, clinical symptoms like weight loss, respiration rate, and temperature were assessed for 3 days in vaccinated and nonvaccinated cotton rats.

**Figure 2:**
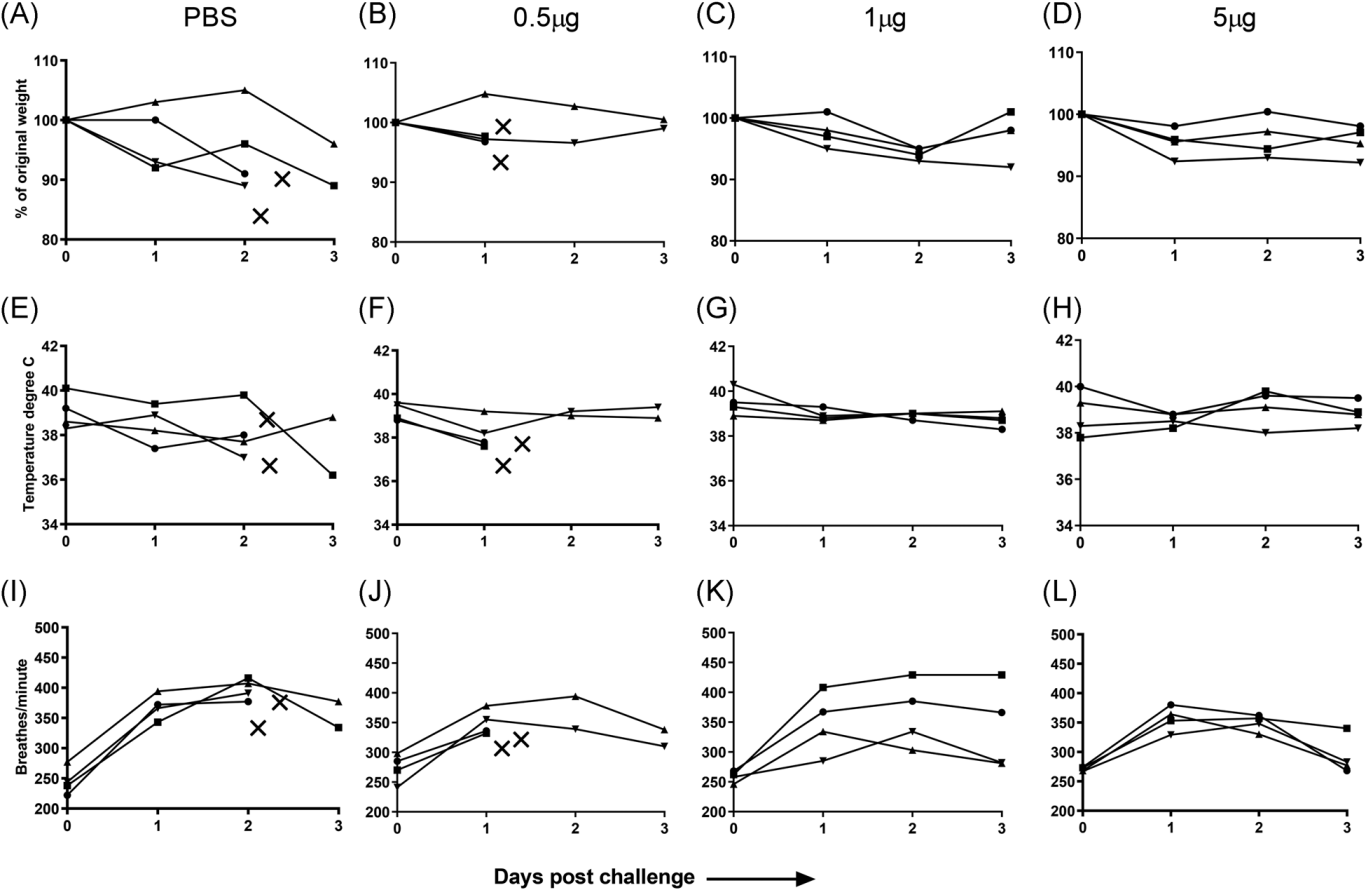
Effect of a single immunization on clinical symptoms after virus challenge. Cotton rats mock‐immunized with PBS or immunized once with 0.5, 1, or 5 µg WIV and challenged on day 30 with 5 × 10^7^ TCID_50_ of homologous A/Cal/Gro were followed for 3 days and weight (A‐D), temperature (E‐H), and respiration rate (I‐L) were recorded. Discontinued line with X symbol indicates dead animal. PBS, phosphate‐buffered saline; WIV, whole inactivated virus

Two animals from the PBS control group were found dead on day three post‐challenge. The two remaining cotton rats from the PBS group did not show considerable weight loss on day 1 post‐challenge, however, their weight reduced over the next 2 days (Figure 2A). Two cotton rats from the 0.5 µg WIV group were found dead on day 2 post‐challenge, the other animals from this group did not show much weight loss (Figure 2B). Animals from the 1 and 5 µg WIV groups lost no or little weight (Figure 2C,D). The animals from the PBS control group and the 0.5 µg WIV group which deceased shortly upon infection showed a slight drop in temperature before death and an additional animal from the PBS group showed a substantial decrease in body temperature on day 3 (Figure 2E). The temperature was not affected by the virus infection in other animals (Figure 2E‐H).

All four cotton rats from the PBS control group presented with a significant increase in respiration rate on day two post‐challenge and then the respiration started to normalize (Figure 2I). Also, all the animals in the 0.5 µg WIV group showed increase in respiration rate on day 2 (Figure 2J). In the 1 µg WIV group, two of the animals demonstrated a marked and sustained increase in respiration rate post‐challenge, the remaining two animals presented with only a moderately increased respiration rate, which returned to normal values by day three (Figure 2K). Cotton rats in the 5 µg WIV group also showed increased respiration on day 1 post‐challenge. However, on day 2, the respiration rate in these animals started to decrease and was significantly lower than the respiration rate in the mock‐immunized control animals on day 2 (*P = .0286). For three of the four animals the respiration rate came back to the baseline on day three (Figure 2L). Hence, the respiration rate was the most sensitive parameter of infection in the used cotton rat model.

In conclusion, immunization with a sufficiently high dose of WIV prevented infection‐induced death of the cotton rats but provided limited protection against clinical symptoms. Some of the control animals and of the 0.5 µg WIV group died rapidly without prior overt signs of distress.

### 3.3 Effect of a single immunization on lung virus titers

**Figure 3:**
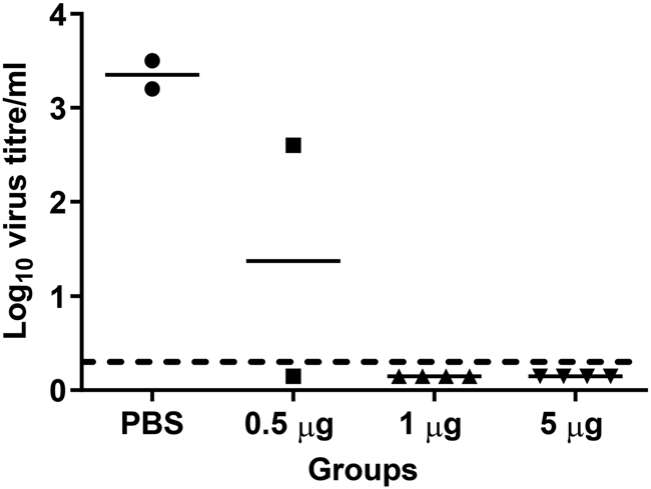
Effect of a single immunization on lung virus titers. The surviving cotton rats from the experiment described in the legend to Figure 2 were killed 3 days post‐challenge and lung virus titers were determined. Virus titers for individual cotton rats and the mean titers per experimental group are depicted. LoD for the virus titers is indicated at 0.3 with a dashed line and negative samples are assigned a value corresponding to half of the LoD. LOD, limit of detection; PBS, phosphate‐buffered saline

To assess the effect of the immunization on virus replication, immunized and PBS‐treated animals were killed 3 days post‐challenge to evaluate lung virus titers. As two animals from each of the PBS treated and the 0.5 µg WIV groups were found dead before day 3, virus titers could not be retrieved from them. The two surviving cotton rats from the PBS group showed titers of 10^3.2^/mL and 10^3.5^/mL, respectively. Of the surviving animals from the 0.5 µg WIV group, one had a virus titer of 10^2.8^/mL while the other had no detectable virus in the lung. Animals vaccinated with 1 or 5 µg WIV were all free of virus in the lungs 3 days post‐infection (Figure 3). No virus was found in the nose of any of the animals. Thus, even a single vaccination with a moderate vaccine dose of 1 µg reliably protected against replicating virus in the lungs upon challenge.

### 3.4 IgG responses after prime/boost vaccination and challenge

**Figure 4:**
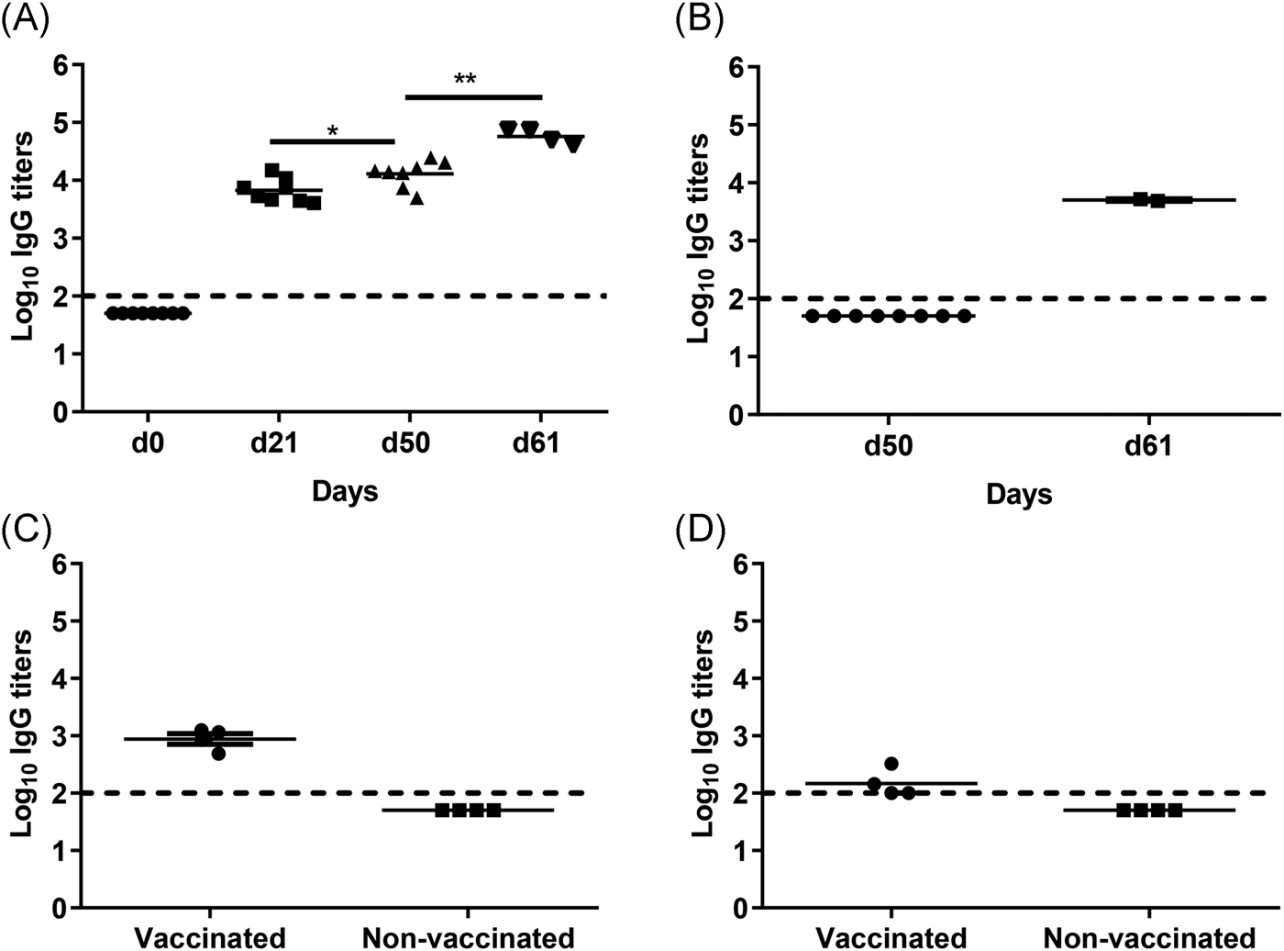
IgG responses after prime/boost vaccination and challenge. Serum samples collected on day 0, after the first vaccination (d21), on the day of challenge (d50), and 10 days post‐challenge (d60) were used for the determination of IgG titers. IgG ELISA was performed for sera of (A) vaccinated cotton rats and (B) nonvaccinated cotton rats. Cross‐reactive IgG against A/PR/8 H1N1 (C) and against X‐31 H3N2 (D) was measured in d50 serum samples. Titers are represented as log_10_ titers with significance *P < .05 and **P < .01. LoD for the IgG titers is indicated at 2 with a dashed line. ELISA, enzyme‐linked immunosorbent assay; IgG, immunoglobulin; LOD, limit of detection; n.d, not determined; PBS, phosphate‐buffered saline; WIV, whole inactivated virus

In the case of a newly emerging virus, naïve individuals are expected to benefit from a booster immunization to achieve protective levels of immunity. To mimic this situation, we immunized cotton rats twice with a moderate dose of 1 µg WIV and challenged them 1 month after the second immunization. To measure the immune response induced by immunization and/or infection, IgG ELISA was performed on serum samples taken from vaccinated as well as mock‐vaccinated cotton rats on the days of immunization (d0, d21), the day of challenge (d50), and 10 days post‐challenge (d60). IgG titers were determined against homologous NIBRG‐121 (Figure 4A). None of the animals showed any influenza‐specific IgG on the day of the first immunization. In line with the first experiment, cotton rats from the WIV‐immunized group developed IgG titers of around 10^3.8^ after the first immunization; these titers increased slightly but significantly after the second vaccination. Interestingly, upon challenge, IgG titers increased further indicating a booster by the infection itself. For nonvaccinated cotton rats, there was no serum IgG on the day of challenge (Figure 4B), but 10 days post‐challenge, virus‐specific IgG was readily detectable in the two surviving animals confirming successful infection.

To assess the cross‐reactive potential of these IgG antibodies, ELISA was performed to measure antibodies against heterologous A/PR/8 H1N1 and heterosubtypic X‐31 H3N2. All cotton rats vaccinated twice with 1 µg WIV showed IgG responses to A/PR/8 but titers were about 1 log lower than those against the homologous virus (Figure 4C). Yet, only two of the four vaccinated cotton rats had IgG cross‐reactive with X‐31 and the titers were much lower than those to the homologous virus (Figure 4D).

### 3.5 Assessment of the functional potential of systemic antibodies by HI and MN upon prime/boost vaccination

**Figure 5:**
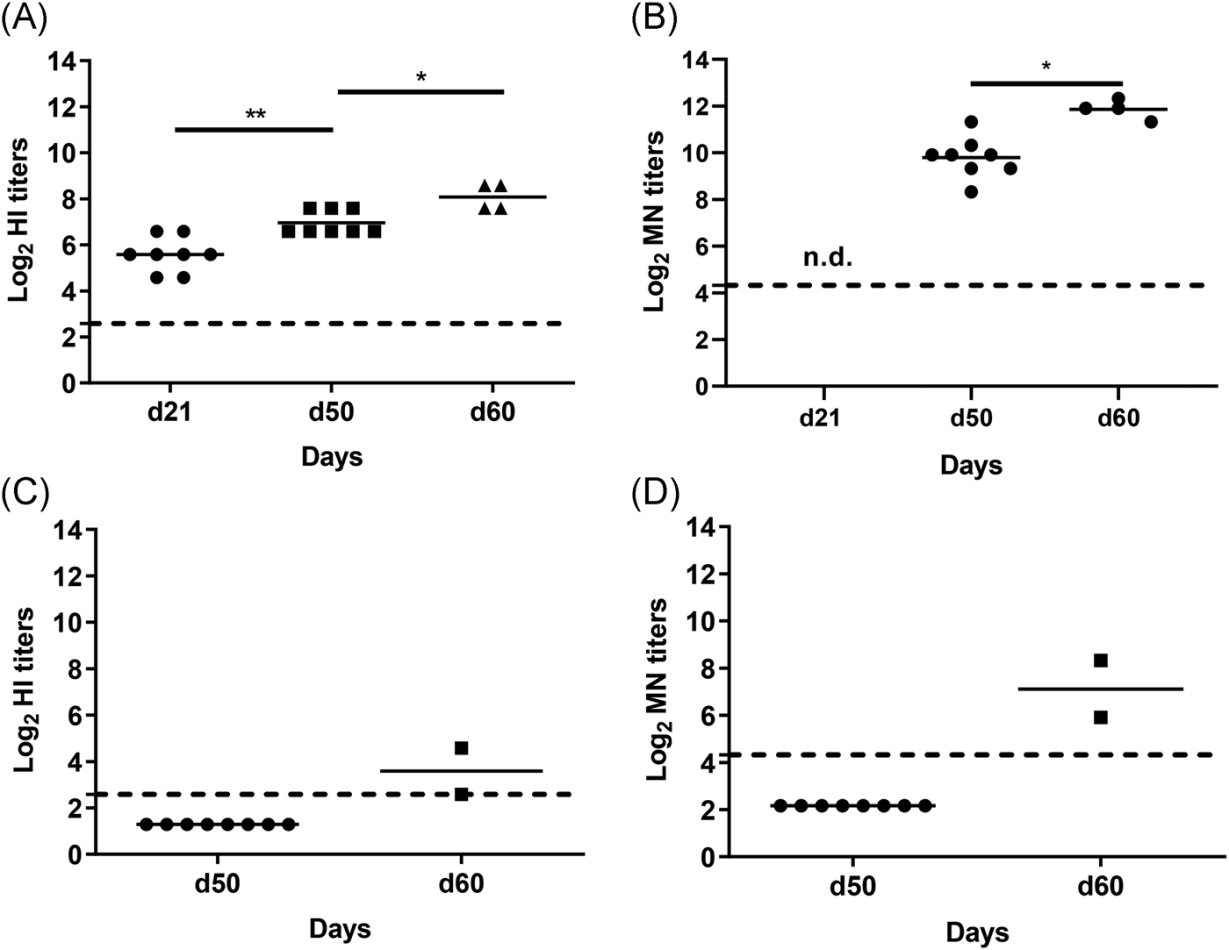
Assessment of the functional potential of systemic antibodies by HI and MN assays. Serum samples were collected at the second vaccination (d21), day of challenge (d50), and 10 days post‐homologous challenge (d60) and functionality of the IgG was measured by HI (A,C) and MN (B,D) assays for vaccinated (A,B) and nonvaccinated cotton rats (C,D). HI and MN antibody titers against NIBRG‐121 virus are represented as log_2_ titers. Significance as *P < .05, **P < .01. LoD for the MN titers is indicated at 4.32 and LoD for the HI titers is indicated at 2.58, both indicated with a dashed line. HI, hemagglutination inhibition; LOD, limit of detection; MN, microneutralization; n.d, not determined

To assess the functional potential of the serum IgG, HI and MN assays were performed using homologous NIBRG‐121. In line with the IgG antibody responses, HI (Figure 5A) and MN (Figure 5B) antibodies were readily detected after two vaccinations with 1 µg WIV and increased further after challenge. For nonvaccinated cotton rats, HI (Figure 5C) and MN (Figure 5D) antibodies were undetectable before challenge but were present 10 days post‐challenge although at lower levels than in vaccinated animals. We also checked for the cross‐neutralizing potential of serum antibodies against A/PR/8 H1N1 and X‐31 H3N2 virus. However, despite the presence of cross‐reacting antibodies detected by ELISA these antibodies could not neutralize the heterologous and heterosubtypic viruses (results not shown).

### 3.6 Effects of prime/boost vaccination on clinical symptoms post‐challenge

As a single immunization with 1 µg WIV completely protected from virus replication but not from clinical symptoms in the first experiment, we next assessed whether the protection provided by WIV could be improved by giving a booster vaccination. For this purpose, in the second experiment, cotton rats were vaccinated twice with 1 µg NIBRG‐121 WIV with a 21‐day interval and were challenged with 1 × 10^7^ TCID_50_ of homologous A/Cal/Gro 30 days later. The lower challenge dose compared with the first experiment was chosen to slow down the disease process. Upon challenge, animals were followed daily for 10 days for changes in weight, temperature and respiration rate.

**Figure 6:**
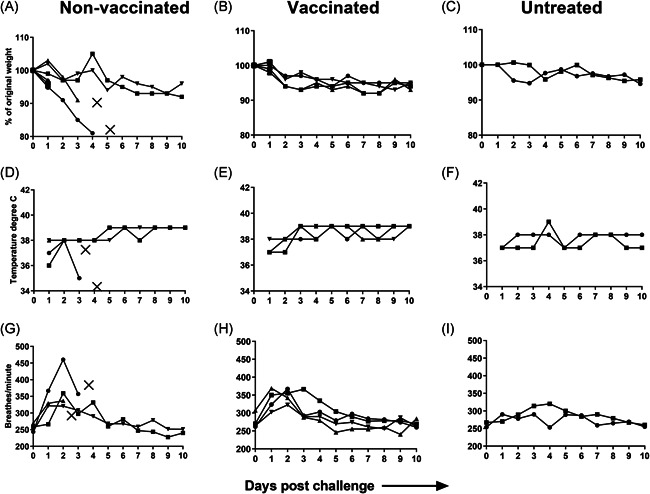
Effects of prime/boost vaccination on clinical symptoms post‐challenge. Cotton rats were injected with PBS, immunized twice with 1 µg WIV, or were left untreated. Thirty days after the second immunization, immunized and mock‐immunized animals were challenged with 1 × 10^7^ TCID_50_ of homologous virus, nontreated animals were again left untreated. After challenge, animals were followed for weight loss (A‐C), change in temperature (D‐F), and respiration rate (G‐I). Discontinued line with X symbol indicates dead animals. PBS, phosphate‐buffered saline; WIV, whole inactivated virus

Despite the fivefold lower virus challenge dose, two of the four mock‐vaccinated cotton rats were found dead in the cage on days 3 and 4 post‐challenge. The remaining two cotton rats from the control group displayed minor weight loss but survived until the end of the follow‐up period (Figure 6A). Cotton rats vaccinated twice with 1 µg WIV lost some weight until day 2 post‐challenge (Figure 6B), thereafter, the weights were stable till sacrifice. Interestingly, two control cotton rats that were neither vaccinated nor challenged also lost also some weight over time (Figure 6C) and did not regain it, which might suggest that daily handling caused a stress response affecting eating or drinking behavior. Temperature was not affected in the infected animals except for one of the mock‐vaccinated cotton rats, which showed a decline in temperature 1 day before death (Figure 6D‐F).

All of the mock‐vaccinated cotton rats showed a significant increase in respiration rate between the day of challenge and day 2 post‐challenge, indicating successful infection (Figure 6G). One of the four cotton rats reached a respiration rate of around 460 and was found dead 2 days later. Cotton rats vaccinated with 1 µg WIV also presented with a significantly higher respiration rate on day 2 post‐challenge as compared with the day of challenge (Figure 6H). There was no statistically significant difference in the day 2 respiration rate between mock‐vaccinated and vaccinated animals in this experiment (*P* = 1.0000). After day 2, the respiration rate started to return to normal. Nontreated cotton rats did not show much change in the respiration rate compared with their baseline respiration rate (Figure 6I).

### 3.7 Effect of prime/boost vaccination on lung virus titers after challenge

**Figure 7:**
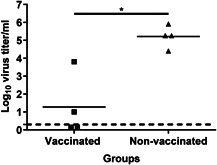
Effect of prime/boost vaccination on lung virus titers after challenge. Thirty days after the second vaccination, cotton rats were challenged with 10^7^ TCID_50_ homologous A/Cal/Gro by i.n. administration. One day post‐challenge, lung virus titers were determined by TCID_50_. Virus titers of individual animals and the mean virus titer per experimental group are depicted. Significance is represented as *P < .05. LoD for the virus titers is indicated at 0.3 with a dashed line. LOD, limit of detection

One day post‐challenge, the peak day of virus in the lungs according to the literature,^20^ half of the animals from the vaccinated and the mock‐vaccinated group were killed to evaluate the viral load in the lungs. All cotton rats from the mock‐vaccinated group had virus in their lungs, with a mean titer of 10^5.51^/mL (Figure 7). In contrast, out of four immunized cotton rats, two did not show detectable virus and the remaining two showed reduced titers (10^1^ and 10^3.8^) as compared with the virus titers in nonvaccinated cotton rats (lowest titer: 10^4.4^). Thus, a booster vaccination with a moderate vaccine dose provided significant protection (*P* = .0294) from early virus replication in the lungs.

### 3.8 Effect of prime/boost vaccination on infection‐related expression of Mx and cytokine genes in the lung

To evaluate the effects of vaccination on expression of infection‐related genes in the lungs upon challenge, qRT‐PCR was performed on messenger RNA (mRNA) isolated from lung tissue; mRNA derived from lungs of nontreated cotton rats was used to set the baseline expression.

**Figure 8:**
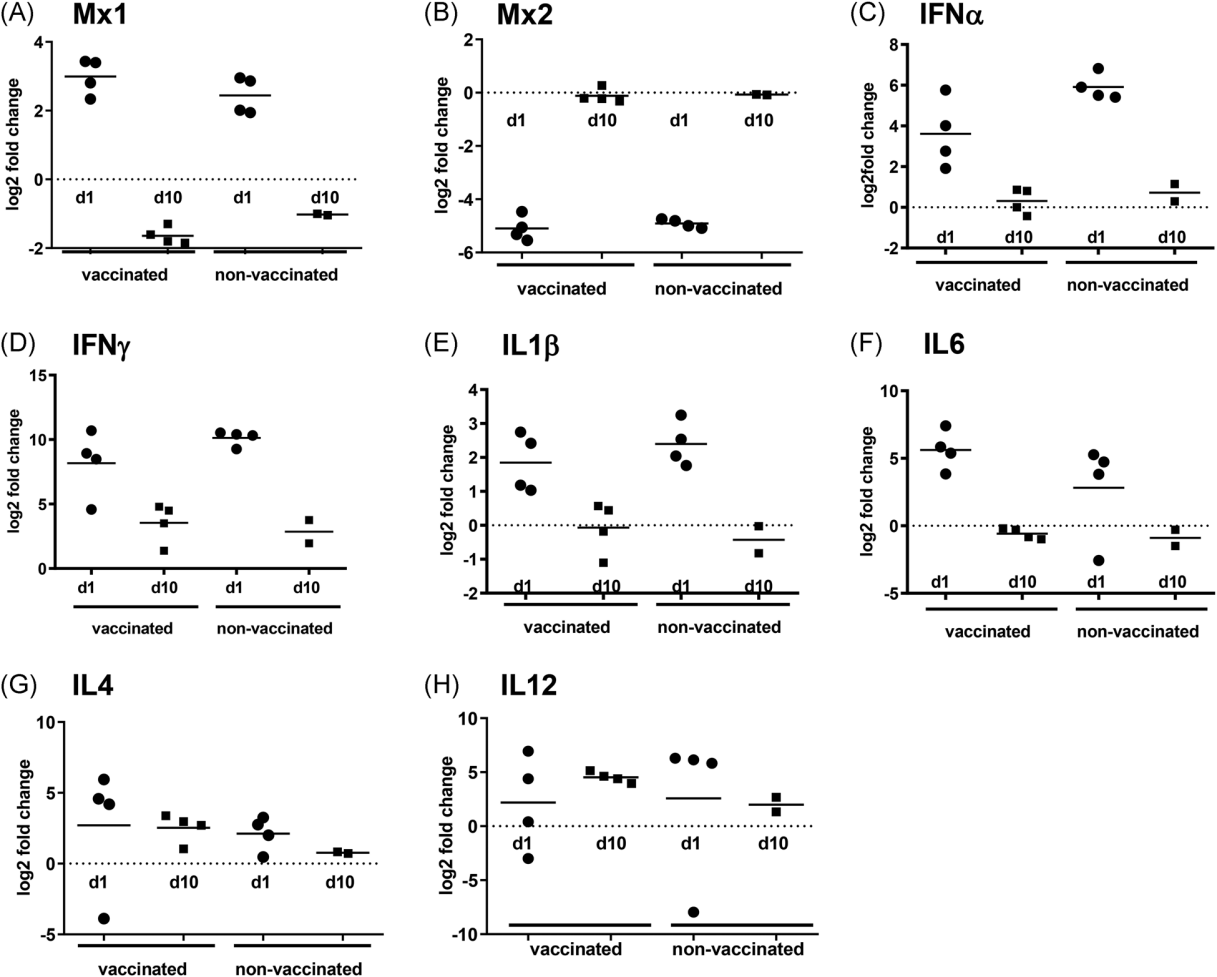
Effect of prime/boost vaccination on gene expression in the lungs upon infection. Cotton rats were (mock‐)immunized, boosted and challenged as previously described. On day 1 and day 10 after virus challenge, expression of cytokine mRNA in lungs of the animals was assessed by qRT‐PCR with gene‐specific primers using the housekeeping gene GAPDH as a reference. GAPDH, glyceraldehyde 3‐phosphate dehydrogenase; mRNA, messenger RNA; qRT‐PCR, quantitative real‐time polymerase chain reaction

Mx proteins can inhibit virus replication and have been described to be strongly induced in the lungs of influenza‐infected cotton rats.^25^, ^26^ Infection clearly led to increased expression of Mx1 1 day post‐challenge in both vaccinated and nonvaccinated cotton rats but levels had returned to normal or even less than normal by day 10 (Figure 8A). In contrast, Mx2 was strongly downregulated (around fivefold) in cotton rats from both groups on day 1 post‐challenge (Figure 8B), but reached baseline levels again by day 10. There was no difference in expression of Mx1 and Mx2 between nonvaccinated and vaccinated animals.

Another response to influenza infection in cotton rats is the upregulation of cytokine expression.^20^ We observed that infection led to increased expression, particularly of IFNα, IFNγ, IL1β, and IL6 on day 1 post‐challenge. By day 10, expression of these cytokines had declined again, though not in all cases, to baseline (Figure 8C‐F). Although expression of the aforementioned cytokines clearly peaked shortly after infection and then declined again, expression of IL4 and IL12 did not show such a trend (Figure 8G,H) but rather showed a moderate but sustained increase. Vaccination did not have significant effects on cytokine expression in the lungs upon infection. Only for IFNα, there was a trend to lower expression in vaccinated animals and IL4 expression was higher in three out of four vaccinated animals as compared with nonvaccinated animals on day 1 post‐challenge.

## 4 DISCUSSION

The aim of this study was to assess the humoral immune responses and the degree of protection (clinical and virological) induced upon use of different influenza vaccination regimens, single immunization with different vaccine doses and prime/boost immunization with a moderate vaccine dose. We demonstrate that all immunized animals developed a humoral immune response against the vaccine strain. A single immunization with a dose more than 0.5 μg antigen as well as prime/boost immunization before virus challenge resulted in reduction of lung virus titer (on day 1 or day 3) and improved survival but had rather moderate effects on clinical symptoms. In particular, increased respiration rate, the most sensitive parameter of infection, was only slightly ameliorated in vaccinated animals. Furthermore, upon prime/boost vaccination, gene expression in the lungs of the vaccinated animals was found to be similar to the gene expression in the lungs of nonvaccinated animals. Although we used outbred cotton rats, we found variation among the animals to be acceptable.

As a challenge virus, we used a clinical isolate of H1N1pdm09 virus. Upon whole respiratory tract challenge, we could readily detect the virus in the lungs of the cotton rats on days 1 and 3 post‐infection, indicating successful infection. Yet, we observed no or very little effect of the infection on weight loss and temperature. This is in line with a study by Blanco et al and our own study, which previously reported that infection with H1N1pdm09 does not cause overt weight loss and/or drop in temperature in cotton rats in contrast to infection with other influenza virus strains.^18^, ^26^ However, H1N1pdm09 infection led to a marked increase in the respiration rate of the animals, which was in line with what we have reported earlier.^18^ In nonvaccinated animals, the peak of increased respiration rate was on day 2 post‐challenge, which correlates with the peak of lung histopathology on day 2 as reported by Blanco et al. In the second study, although we used five times less virus, interestingly, an increased respiration rate was also observed in vaccinated animals (upon prime‐boost vaccination). This indicates that vaccinated animals still developed some disease symptoms despite the fact that lung virus titers were strongly reduced or even below detection limit. We attribute this to the moderate dose of vaccine used in this study.

When investigating gene expression in the lungs of infected cotton rats, we found upregulation of Mx1 mRNA as well as of mRNAs encoding IFNα, IFNγ, IL1β, and IL6, indicating induction of an antiviral response. This result corroborates the findings from earlier studies, which report IFN expression in influenza‐infected cotton rats to correlate with replicating virus in the lung and to mediate antiviral responses by induction of Mx1 genes and other response modifiers.^21^, ^26^, ^27^, ^20^, ^25^, ^28^ Many of these cytokines are expressed early upon infection and at this time point virus replication peaks as well in cotton rats.^20^, ^28^ And that is why day 1 post‐challenge was an interesting time point to assess cytokine expression. In contrast to earlier reported results, we observed a strong downregulation of Mx2 mRNA on day 1 post‐challenge in both vaccinated and nonvaccinated animals.^25^ The reason for this discrepancy is unknown but could possibly be related to the virus strain used (A/Wuhan.359/95 H3N2 in the previous study, H1N1pdm09 here). Mx2 is not involved in the response to influenza virus infection^27^, ^29^, ^30^ and its downregulation might thus simply be the result of a general shut down of host gene expression as observed in influenza‐infected cells.^31^, ^32^ Levels of expression of Mx1 and the mentioned cytokines were similar for naïve and vaccinated animals. This further corroborates that for the vaccination regimens investigated in this current study, sterilizing immunity could not be achieved, which is why we do not see a difference in the expression pattern of these cytokines between vaccinated and nonvaccinated animals. We attribute this to the challenge virus.

When evaluating the effect of vaccination, we observed that a single i.m. injection of WIV even with a low dose of 0.5 µg was sufficient to induce humoral immune response and to reduce (0.5 µg) or abolish (1 and 5 µg WIV) virus replication in the lungs. A prime/boost vaccination regimen with a moderate antigen amount of 1 µg increased the serum antibody titers significantly. Interestingly, the HAI titers were even of the same magnitude as the ones induced by two immunizations with 15 µg WIV in our previous study.^18^ Although there was a disconnect observed among the titers of IgG, HAI and MN antibodies, the overall trend of responses was similar. However, in contrast to the previously published study, we did not observe complete protection against clinical symptoms in this current study.

There are several studies evaluating the effect of influenza vaccination in cotton rats; however, these studies do usually not investigate clinical symptoms in detail. A study by Yim et al^12^ showed that a single i.m. vaccination with a low dose of trivalent inactivated vaccine, FluLaval, was efficacious in inducing antibody responses in cotton rats and preventing virus replication in the lungs but a booster vaccination was needed to protect also from virus replication in the nose. Unfortunately, no data on disease symptoms are provided. In a study by Crosby et al, a single vaccination with a low dose of an HA‐expressing AAV vaccine could only partly protect the animals from lung virus replication in spite of effectively inducing anti‐HA antibodies.^16^ These and our own observations suggest that complete protection of cotton rats against influenza infection might require more than HA‐specific antibodies alone. For example, it has been reported that passively transferred antibodies against M2e can partly protect cotton rats against influenza‐induced tachypnea indicating a role for anti‐M2e in protection.^11^ It might be that the low or moderate doses of vaccine as used in the current study were insufficient in inducing anti‐M2e antibodies and other so far unidentified immune mechanisms and thus could not confer complete protection.

There are certain limitations of this study which can be overcome in future studies. Our study focusses on the effects of vaccination‐induced humoral immunity. However, the effect of different vaccination regimens on T cell immunity would also be interesting to explore. Furthermore, for this proof‐of‐concept study, we used a sample size of four animals per group like many other cotton rat studies do. Yet, to accommodate for inter‐animal variation and possible loss of animals, in future studies with outbred cotton rats, larger group sizes should be used. To improve the immune response and in turn the protection, vaccine dose could be increased or other routes of vaccination or inclusion of adjuvants could be tested.

In conclusion, outbred cotton rats can be a good model for influenza infection and vaccine evaluation studies, as infection with clinical isolates is possible without preadaptation and immune responses can be easily induced by vaccination. Protective effects of vaccination should be investigated by assessing not only virus titers but also by evaluating clinical symptoms, especially tachypnea, as a particularly sensitive parameter. Doing so enables to mimic complete and partial protection from influenza by using specific vaccination regimens. Being small, affordable and susceptible to clinical influenza virus isolates, cotton rats have their place between the clinically less relevant inbred mouse model and the expensive ferret model for the evaluation of influenza vaccine candidates.

## CONFLICT OF INTERESTS

The authors declare that there are no conflict of interests.

## AUTHOR CONTRIBUTIONS

Y.B. and A.H. conceived and designed the experiments and analyzed the data and wrote the manuscript. H.G.N. provided the clinical isolate of influenza virus for the challenge studies. Y.B., T.M., and W.D. performed the animal experiments. Y.B. and J.d.V.‐I. performed the assays. All authors have approved the manuscript.

## Supporting information

Supporting information

## Data Availability

The data generated within the study is shown in this manuscript. Any raw data or analysis would be available from the corresponding author upon request.
