# Supplementary material for: Characterization of humoral immune responses and degree of protection induced by influenza vaccine in cotton rats: Effects of low vaccine dose and single vs booster vaccination
Source: Immun Inflamm Dis. 2020 Apr 22;8(3):279–91. doi: 10.1002/iid3.303 (PMC7416045; doi:10.1002/iid3.303)
Supplement: Supplementary file 1 — Supporting information [file IID3-8-279-s001.docx]

|  | **FW Primer 5'-->3'** | **BW Primer 5'-->3'** | **bp product** |
| --- | --- | --- | --- |
| **GAPDH** | GAT-GCC-CCC-ATG-TTT-GTG-AT | AGA-CAC-GTT-GGG-GGT-AGG-AA | 318 |
| **IFN-γ** | GAA-CAG-TCC-GCT-GTT-TCT-GC | ACT-GCA-GCC-ATG-TGA-GAA-CT | 470 |
| **IL-4** | GTA-CCG-GGA-ACT-GTA-CTC-ACG | AGA-CTG-CTG-ATG-CCC-CTG-TA | 274 |
| **IL-6** | AGG-ATC-CGG-GTA-AAA-CAG-CC | GCC-GAG-TAG-ACC-TCA-TGG-TG | 156 |
| **Mx-1** | AGT-GTG-GAA-AGA-GCA-TCG-GG | CAG-GAG-CCG-GCT-ACA-GTT-AG | 131 |
| **Mx-2** | AGC-CCT-GTT-TCA-GTC-AGC-TC | ATG-GCT-CTC-AGG-GAG-TGG-AT | 152 |
| **IL-1-b** | TCT-TTG-AGG-TTG-ACG-AGC-CC | GAG-TGA-CAC-CAC-CTG-CTT-GA | 456 |
| **IL-12-p-40** | TCA-GCA-CGG-ACC-TGA-AGT-TT | GGT-GGG-TCC-GGT-TTG-ATG-AT | 274 |
| **IFN-α** | TGC-TGG-CAA-GAT-GGA-GTG-AG | TGA-TGG-CAT-ATC-ATG-GGT-ATC-TGT | 326 |

**Supplementary table 1. Primer summary**
